# Genome Analysis of *Shigella flexneri* Serotype 3b Strain SFL1520 Reveals Significant Horizontal Gene Acquisitions Including a Multidrug Resistance Cassette

**DOI:** 10.1093/gbe/evz026

**Published:** 2019-02-01

**Authors:** Pawan Parajuli, Lachlan P Deimel, Naresh K Verma

**Affiliations:** Division of Biomedical Science and Biochemistry, Research School of Biology, The Australian National University, Canberra, Australian Capital Territory, Australia

**Keywords:** *Shigella flexneri*, hybrid sequencing, complete genome, bacteriophage, multidrug resistance

## Abstract

*Shigella flexneri* is a major etiological agent of shigellosis in developing countries, primarily occurring in children under 5 years of age. We have sequenced, for the first time, the complete genome of *S. flexneri* serotype 3b (strain SFL1520). We used a hybrid sequencing method––both long-read MinION Flow (Oxford Nanopore Technologies) and short-read MiSeq (Illumina) sequencing to generate a high-quality reference genome. The SFL1520 chromosome was found to be ∼4.58 Mb long, with 4,729 coding sequences. Despite sharing a substantial number of genes with other publicly available *S. flexneri* genomes (2,803), the SFL1520 strain contains 1,926 accessory genes. The phage-related genes accounted for 8% of the SFL1520 genome, including remnants of the Sf6 bacteriophage with an intact *O*-acetyltransferase gene specific to serotype 3b. The SFL1520 chromosome was also found to contain a multiple-antibiotic resistance cassette conferring resistance to ampicillin, chloramphenicol, streptomycin, and tetracycline, which was potentially acquired from a plasmid via transposases. The phylogenetic analysis based on core genes showed a high level of similarity of SFL1520 with other *S. flexneri* serotypes; however, there were marked differences in the accessory genes of SFL1520. In particular, a large number of unique genes were identified in SFL1520 suggesting significant horizontal gene acquisition in a relatively short time period. The major virulence traits of SFL1520 (such as serotype conversion and antimicrobial resistance) were associated with horizontal gene acquisitions highlighting the role of horizontal gene transfer in *S. flexneri* diversity and evolution.

## Introduction

*Shigella* species are the Gram-negative facultative anaerobic bacteria that cause shigellosis, characterized by acute colonic and rectal mucosal inflammation leading to fever, abdominal cramps, and bloody mucoid stools (Jennison and Verma 2004). It is estimated that at least 190 million shigellosis cases occur annually with 70,000 resulting in death (Pires et al. 2015). Although no individual is immune to shigellosis, the morbidity and mortality are highest among children <5 years of age (Niyogi 2005).

The serology of *Shigella* is dependent primarily on its O-antigen of the outer membrane lipopolysaccharide (Ewing and Lindberg 1984). *Shigellaflexneri*, the most common species of *Shigella*, is known for having a number of serologically distinct strains, which arise from the alteration of the O-antigen backbone comprised of →2)-α-L-Rha*p*^III^-(1→2)-α-L-Rha*p*^II^-(1→3)-α-L-Rha*I*^I^-(1→3)-β-d-Glc*p*NAc-(1→ (Kenne et al. 1977). The patterns of glucosylation, acetylation, and/or phosphorylation to one or more sugars along the backbone distinguishes O-antigens and, therefore, serotypes (Allison and Verma 2000).

The population-wide variation in O-antigen structure presents challenges in the development of broad-spectrum immunity (Theillet et al. 2011). Vaccine strategies in the past have combined the historically more prominent serotypes, 3a and 6, with the inclusion of a 2a strain to promote broader immunity (Walker 2015; DeLaine et al. 2016). As the microbiology and immunology communities strive to generate a sufficiently broad vaccine to cover major *S. flexneri* serotypes, it is critical that the uniqueness of serotypes as well as cross-protection patterns are better understood, especially with respect to variation in both functionality and genomics.

**Fig. 1.**
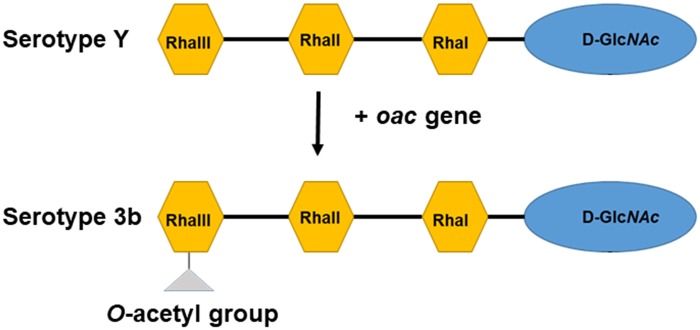
—Schematic representation of *S. flexneri* serotype Y and *S. flexneri* serotype 3b O-antigen. Each tetrasaccharide unit has one *N*-acetyl glucosamine (D-Glc*NAc*) and three rhamnose sugars (Rha).

One example of a *S. flexneri* serotype that has been the subject of limited studies is serotype 3b; despite being one of the most common serotypes in Asia, accounting for ∼28% of the incidence in Thailand, little is known about the genome organization and virulence features of 3b, including cross-protection (Chompook et al. 2005). This serotype is characterized by the presentation of an *O*-acetyl group (group antigen 6) on the rhamnose III of the O-antigen, with no additional glucosyl or phosphoethanolamine group along the oligosaccharide (fig. 1) (Ewing and Carpenter 1966). The molecular characterization of bacteriophage SF6 encoded *O*-acetyltransferase (*oac*) gene from serotype 3b was first elucidated by Verma et al. (1991). The *S. flexneri* serotype 3b strain SFL1520 has been shown to be highly virulent based on in vitro and in vivo studies using *Caenorhabditis elegans* and the murine pulmonary model (George, Behm, et al. 2014; George, Mathesius, et al. 2014). Despite the unique O-antigen modification and virulence being well elucidated, an analysis of its genome is absent from the literature. Because of the highly repetitive genome of *Shigella* chromosome, sequence data generated by short-read sequencing technologies impair genome assembly (Scheibye-Alsing et al. 2009). In this study, we report the first complete genome analysis of *S. flexneri* serotype 3b strain SFL1520 using both long-read MinION Flow (Oxford Nanopore Technologies) and short-read MiSeq v3 300-bp (Illumina) sequencing platforms. The availability of the complete genome of SFL1520 and subsequent genome analysis will provide further insights into its genome signatures, including virulence, antibiotic resistance, and evolution.

## Materials and Methods

### Bacterial Strain and Genome Sequencing

The *S. flexneri* serotype 3b strain SFL1520 (also known as strain K-1770) was clinically isolated and kindly provided by K. A. Talukder from the International Centre for Diarrhoeal Disease Research, Bangladesh. The strain SFL1520 was grown aerobically (180 rpm) at 37 °C in Luria Bertani broth. The antibiotic susceptibility pattern of SFL1520 was determined using the disk diffusion method (Kirby-Bauer) (Cavalieri et al. 2005). We tested SFL1520 for resistance against a range of modern antibiotics (Oxoid, United Kingdom), including ampicillin (10 µg), cefoxitin (30 µg), chloramphenicol (30 µg), erythromycin (30 µg), kanamycin (30 µg), nitrofurantoin (300 µg), penicillin (1 U), tetracycline (30 µg), streptomycin (10 µg), and trimethoprim/sulfamethoxazole (1.25/23.75 µg).

The bacterial DNA was extracted using the Genome Tip 100/G (Qiagen) according to the manufacturer’s instructions. The Rapid Sequencing Kit SQK-RAD004 (Oxford Nanopore Technologies) was used for the library preparation and subjected to the MinION Flow cell (R9.4, Oxford Nanopore Technologies) for sequencing. The Nextera XT DNA library preparation kit (Illumina) was used for MiSeq v3 300-bp paired-end sequencing.

### Genome Assembly

The raw fast5 files from the MinION flow were base-called using Albacore v2.0.1 (Oxford Nanopore Technologies). The adapters were removed using Porechop (https://github.com/rrwick/Porechop; last accessed March 2018). The quality of MiSeq reads was assessed using FastQC v0.11.5 (Andrews et al. 2016) and trimmed accordingly using Trimmomatic v0.36 (Bolger et al. 2014). The MinION long reads and MiSeq short reads were used to carry out hybrid assembly using Unicycler v0.3.1 (Wick et al. 2017) and further improved by using Pilon (Walker et al. 2014). The circularization of the genome was achieved by manual comparison and was confirmed by remapping of sequence reads back to the assembly using Burrows–Wheeler Aligner (Li 2013), SAMtools (Li et al. 2009), and IGV (Thorvaldsdottir et al. 2013).

### General Sequence Analysis

The genome of SFL1520 was annotated using Rapid Prokaryotic Genome Annotation (Prokka) (Seemann 2014). The annotated genome was then subjected to the PHAge Search Tool Enhanced Release (PHASTER) (Arndt et al. 2016) to identify regions of the SFL1520 genome that correspond to the genomic regions of bacteriophages within the database. The SFL1520 genome was compared with other publicly available *S. flexneri* strains using Mauve (Darling et al. 2004) to identify genomic blocks whereby translocation, inversion, and other major genomic events might have occurred. ISsaga was used to predict the number of insertion sequence (IS) elements in the compared genomes (Varani et al. 2011). The presence of genomic island was investigated using IslandViewer 4 tool (Bertelli et al. 2017). The image files were generated using SnapGene Viewer (Version 3.3.4), CGView Server (Grant and Stothard 2008), and the Artemis Comparison Tool (Carver et al. 2005).

### Pangenome and Phylogenetic Analysis

The phylogenetic and pangenome analyses were performed on the genomes that are publicly available and were all reannotated using prokka (Seemann 2014) to reduce the effects of biases. The genes that were common in all compared strains (core genes) and accessory genes were extracted using Roary (Page et al. 2015). The BLAST analysis was performed to identify the potential source of unique genes present in SFL1520 using PHASTER (Arndt et al. 2016), ISsaga (Varani et al. 2011), and ACLAME (Leplae et al. 2010) databases. The contiguity of unique genes in the genome was explored to infer the number of horizontal gene acquisition events that might have occurred in SFL1520 (Grana-Miraglia et al. 2017). The core gene alignment was used to construct a phylogenetic tree using IQ-TREE (Nguyen et al. 2015) based on the model determined by ModelFinder (Kalyaanamoorthy et al. 2017). The bootstrap analysis was conducted using 1,000 randomizations. The tree was visualized using FigTree v1.4.3 (Rambaut 2016).

## Results

### Genome Data

The number of MinIon reads passing the base calling was 211,358 accounting 2,162,407,181 bp (470× coverage) with an N50 read length of 33,730 bp and longest read length of 499,947 bp. Similarly, MiSeq generated 2 × 847,284 paired-end reads of 300 bp (110× coverage). The hybrid assembly generated a single contiguous bacterial chromosome of 4,575,536 bp.

### General Features

The 4,575,536-bp bacterial chromosome of *S. flexneri* serotype 3b strain SFL1520 included 4,729 coding sequences (CDS), 94 tRNA sequences, and 22 rRNA sequences. The guanine and cytosine (GC) content of the genome found to account for ∼50.9% (2,328,626 of 4,575,536 bp). A comparison of the genome features of SFL1520 with other strains of *S. flexneri* showed a highly conserved composition and genome size (table 1).

**Table 1:** Features of Available *Shigellaflexneri* Genomes

Serotype	Strain	Length (bp)	GC (%)	CDS	tRNA	Accessory Genes^a^	Unique Genes^b^	IS Elements
1c	Y394	4,584,634	50.9	4,699	108	1,896	104	152
2a	2457T	4,599,354	50.9	4,709	101	1,906	26	79
2a	301	4,607,202	50.9	4,715	96	1,912	56	68
2a	981	4,661,157	50.9	4,788	106	1,985	13	27
2a	NCTC1	4,526,576	50.9	4,621	97	1,818	14	28
3b	SFL1520	4,575,536	50.9	4,729	94	1,926	201	355
4c	1205	4,683,636	50.9	4,823	106	2,020	13	49
5b	8401	4,574,284	50.9	4,668	98	1,865	100	246
Xv	20021017	4,650,856	50.9	4,781	103	1,978	22	62
Y	2003036	4,595,814	50.9	4,714	98	1,911	39	60
Yv	Shi06HN006	4,620,903	50.9	4,761	98	1,958	40	56

a Accessory genes refer to genes present that are not a part of the “core genome”; the number of total CDS minus the number of genes present in all strains (core genes).

b Unique genes refer to genes which are present only in that strain (with a 95% identity cut-off).

### Genome Rearrangements

**Fig. 2.**
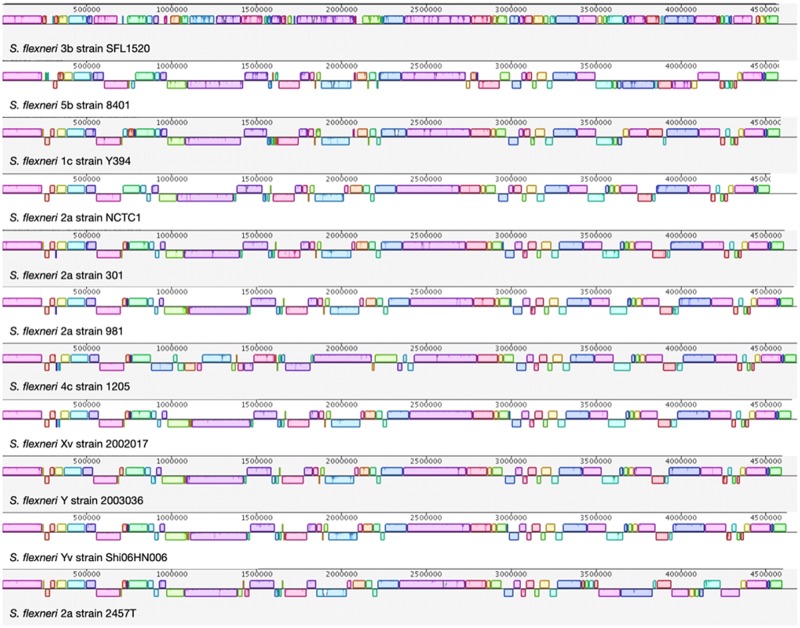
—Whole-genome alignment of *S. flexneri* strains. The horizontal panels represent the genome sequences from top to bottom: *S. flexneri* 3b strain SFL1520, *S. flexneri* 5b strain 8401, *S. flexneri* 1c strain Y394, *S. flexneri* 2a strain NCTC1, *S. flexneri* 2a strain 301, *S. flexneri* 2a strain 981, *S. flexneri* 4c strain 1205, *S. flexneri* Xv strain 20021017, *S. flexneri* Y strain 2003036, *S. flexneri* Yv strain Shi06HN006, and *S. flexneri* 2a strain 2457T. Each colored block refers to a shared synteny among the compared strains. Blocks above and below their respective line depict the orientation of the genomic region with respect to SFL1520. The genomes were added sequentially for comparison based on the phylogenetic distances.

**Fig. 3.**
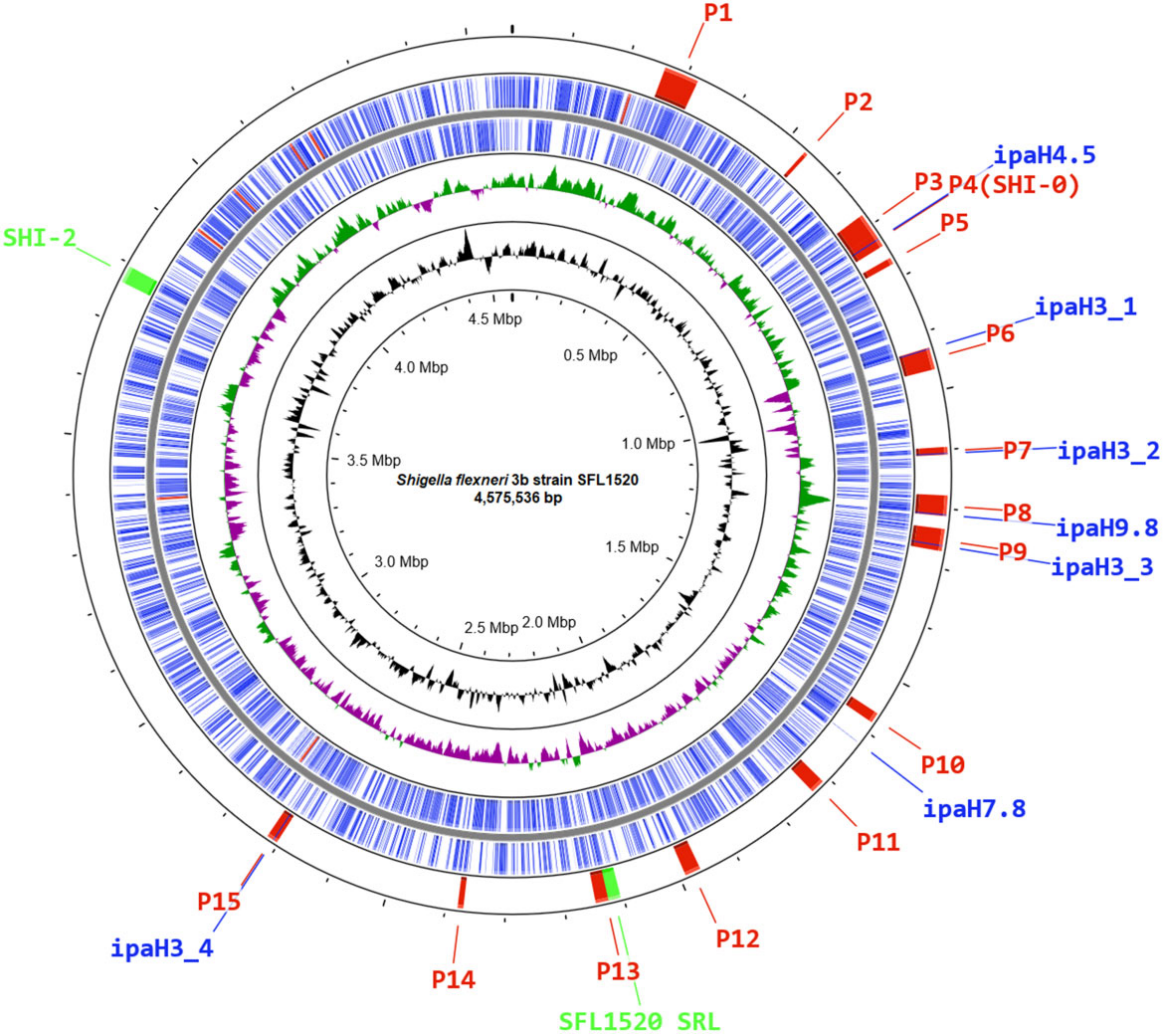
—Schematic circular genome map of *S. flexneri* 3b strain SFL1520. The outermost ring shows the location of bacteriophage regions (P1–P15; red), SFL1520 SRL and SHI-2 (Green) and *ipaH* genes (Blue). The second and third rings depict ORFs of SFL1520 encoded by leading and lagging strands with color codes: CDS (blue) and rRNA (red). The green and purple colors in the fourth ring denote the positive and negative GC skew (G–C/G+C), respectively. The fifth ring in black shows the deviation from average percentage GC content. The innermost ring represents the nucleotide position in the genome.

Whole-genome alignment was conducted between SFL1520 and ten other publicly available strains of *S. flexneri* (supplementary table S1, Supplementary Material online). We identified homologous genomic blocks common to the strains where there existed a high similarity between the strains (fig. 2). The results indicate that there has been a considerable number of genomic shuffling and recombination events among different serotypes and strains of *S. flexneri*. Although the overall GC content is conserved in SFL1520 with 50.9% as in all complete *S. flexneri* genomes, the GC content and skew varied within the genome of SFL1520 suggesting recent acquisitions of genes from distant organisms (fig. 3).

### Mobile Genetic Elements and Pathogenicity Islands

We identified 15 phage regions, cumulatively representing 385 kb (∼8% of the entire bacterial chromosome). The location of each of these phage regions has been shown in figure 3. The regions identified as containing phage or phage-like genes had mostly undergone significant gene deletions, resulting in cryptic prophages.

**Fig. 4.**
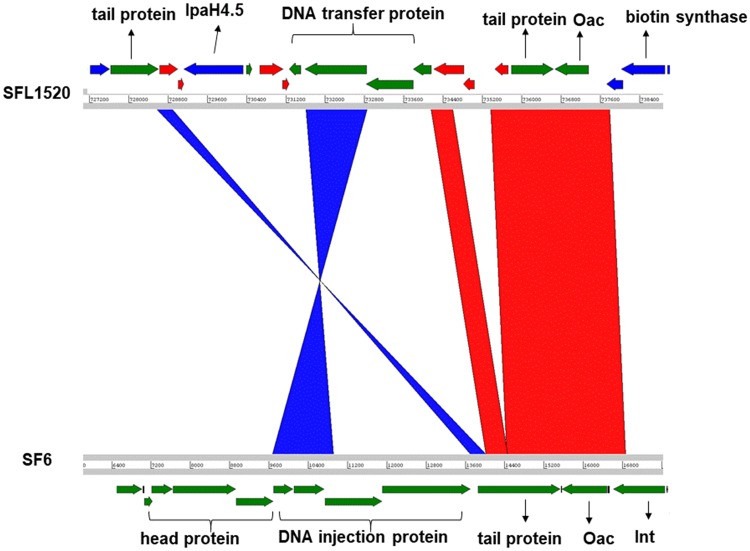
—Comparison of the *oac* gene region of SFL1520 (SHI-O PAI) and SF6 bacteriophage. The genomes are depicted as horizontal gray lines interspersed with regions of collinear (red) and inverted (blue) synteny. The direction of arrows indicates the open reading frames with color code: green, bacteriophage genes; red, IS elements/transposases; and blue, bacterial genes.

On the fourth phage region, there were remnants of the Sf6 bacteriophage including an intact *oac* gene which encodes for *O*-acetyltransferase and is responsible for conferring the 3b serotype (fig. 4). However, there was no complete Sf6 prophage found in this region or throughout the SFL1520 bacterial chromosome.

A total of 355 IS elements were predicted to be present in SFL1520 covering ∼7% (306,441 of 4,575,536 bp) of the total genome; the family IS1 was identified as being the most common, accounting for ∼43% of IS elements (152 of 355 open reading frames [ORFs]).

**Fig. 5.**
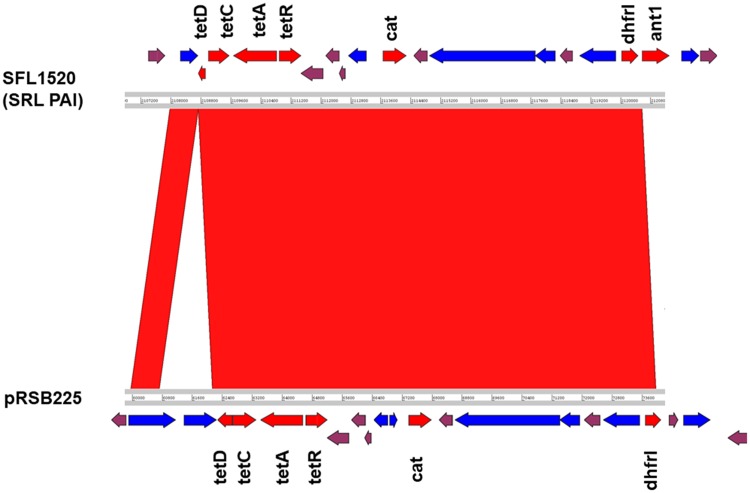
—Comparative analysis of SFL1520 SRL PAI with plasmid pRSB225. The compared genomes are depicted as horizontal gray lines with numbers indicating the corresponding genomic positions. The red shadings between the two genomes indicate the collinear synteny of homologous regions. The arrows indicate the open reading frames in the compared genomes: antibiotic resistance genes (red), IS elements/transposases (blue), and plasmid genes (plum). The original sequence of pRSB225 has been reverse complemented for clarity.

There are four distinct *Shigella* pathogenicity islands (PAIs) namely SHI-0, SHI-1, SHI-2, and invasion plasmid antigen (*ipaH*) islands distributed throughout the genome of recent *S. flexneri* isolates (Ingersoll et al. 2002; Baker et al. 2014). Besides the serotype converting locus (SHI-0), SFL1520 also contains the SHI-2 PAI which is ∼28-kb region comprising genes encoding an aerobactin operon, a colicin V immunity gene, multiple transposases, and several hypothetical genes (Vokes et al. 1999). The genes in SHI-2 PAI include the aerobactin operon consisting of four genes *iucA–D* encoding a siderophore system that allows *Shigella* to uptake iron from the host cell, and *iutA* encodes a bacterial receptor for the siderophore complex (Moss et al. 1999). This region was found in *selC* tRNA locus with similar gene arrangement, reported previously (Moss et al. 1999; Vokes et al. 1999). However, SFL1520 lacks SHI-1 pathogenicity Island characterized in *S. flexneri* serotype 2a consisting of genes *sigA, pic, setA*, and *setB* (Rajakumar et al. 1997). The SFL1520 possesses seven invasion plasmid antigen (*ipaH*) family genes: ORF759 (*ipaH4.5*), ORF952 (*ipaH3*), ORF1162 (*ipaH3*), ORF1294 (*ipaH9.8*), ORF1367 (*ipaH3*), ORF1769 (*ipaH7.8*), and ORF2937 (*ipaH3*) distributed throughout its chromosome (fig. 3). The *ipaH* family genes are present on both the virulence plasmid and the chromosome of *S. flexneri*. The chromosome-encoded IpaH proteins are secreted via type III secretion system and act as effectors to modulate the host inflammatory responses (Ashida et al. 2007). We also identified a 20.4-kb genomic island encoding the multidrug resistance genes *tetDCAR, cat, dhfrI*, and *ant1* (streptomycin 3″-adenylyltransferase) conferring resistance to tetracycline, chloramphenicol, trimethoprim, and streptomycin (fig. 5). The observed phenotype was consistent with the antibiotic resistance genes present in the SFL1520 chromosome. The multidrug resistance gene cassette with similar arrangement has also been previously reported in *Shigella* resistance locus (SRL) PAI of *S. flexneri* serotype 2a strain YSH6000 (Luck et al. 2001), *S. flexneri* serotype Xv strain 2002017 (Ye et al. 2010), and an *Escherichiacoli* plasmid, pRSB225 (Wibberg et al. 2013).

### Pangenome and Phylogeny

The pangenome of the *S. flexneri* was determined by analyzing SFL1520 along with ten other complete genomes of *S. flexneri*. This analysis identified a *S. flexneri* pangenome of 6,056 homologous groups with 2,803 core genes. The unique gene frequencies were found to be variable throughout different *S. flexneri* strains (range 13–201), with SFL1520 having by far the largest number of unique genes (201). This suggests that the SFL1520 accessory genome is distinct from other strains (table 1). These unique genes were associated with 128 horizontal gene acquisition events based on the assumption that two or more genes clustered together might have acquired via same gene transfer event. Further, the BLAST searches of these unique genes revealed that all of these genes were associated with mobile genetic elements such as transposons, phages, and plasmids (supplementary fig. S1, Supplementary Material online).

**Fig. 6.**
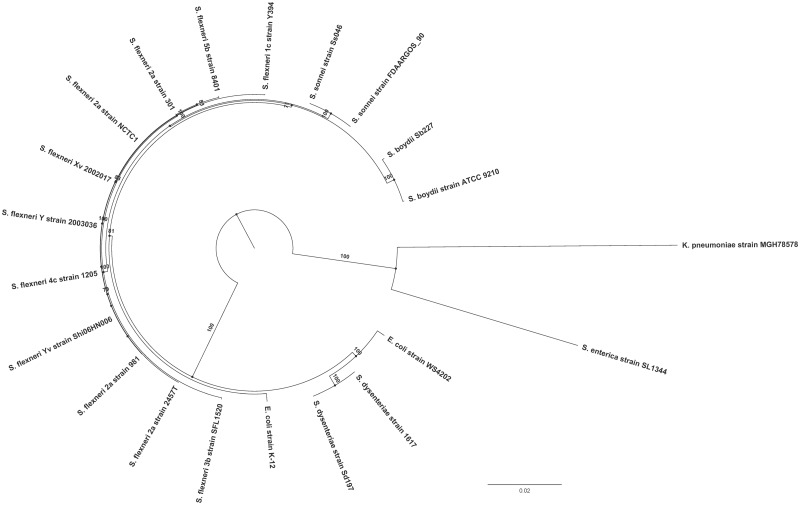
—Phylogeny of *S. flexneri.* The maximum likelihood tree using core genes of the representative *S. flexneri* strains and other *Shigella* species rooted using members of other genera. The numbers indicate bootstrap support values >70 (1,000 pseudoreplicates). The scale bar represents substitutions per site.

The phylogenetic analysis was conducted using 330 genes that were identified as being common to all the compared *S. flexneri* genomes (*n *= 11) as well as broader *Shigella* species and strains representative of *E.coli*, *Klebsiella pneumoniae*, and *Salmonella enterica* (*n* = 10) (supplementary table S1, Supplementary Material online). The maximum likelihood tree was constructed using the general time-reversible model with free rate model (GTR+R3). All *S. flexneri* strains were found to cluster closely together. More distantly were other *Shigella* species, forming a clade with the *S. flexneri* strains. The *Shigella* species, in addition to the *E. coli* strains, formed one of the two main phylogroups, with *S. enterica* and *K. pneumoniae* forming the other as expected (fig. 6). This phylogenetic tree is consistent with *S. flexneri* phylogenetic trees reported previously (Parajuli et al. 2017).

The 2,803 *S. flexneri* core genes were used to find the relatedness of SFL1520 with other *S. flexneri* strains. The *S. flexneri*-specific phylogenetic tree was developed using the transition model (TIM) allowing for a proportion of invariable sites (TIM+I).

The phylogenetic tree based on core genome alignment of *S. flexneri* strains revealed two main phylogroups, with SFL1520 (3b) clustering closely with 8401 (5b) and, more distantly, with Y394 (1c), NCTC1 (2a) and 301 (2a) with well-supported bootstrap values (1,000 pseudoreplicates) (supplementary fig. S2, Supplementary Material online).

## Discussion

We have sequenced, for the first time, the complete genome of *S. flexneri* serotype 3b (strain SFL1520). Here, we aimed to investigate the genomic features and virulence signatures of SFL1520 and to compare its genome with other publicly available *S. flexneri* genomes. Analysis of the SFL1520 bacterial chromosome revealed significant prevalence of mobile elements, including regions of phage genes, IS elements, and PAIs. The phage genes were clustered in 15 regions across the bacterial chromosome and collectively account for 8% of the SFL1520 bacterial chromosome. Of particular interest was the fourth phage region, which contained remnants of bacteriophage Sf6—in particular, the complete *oac* gene, encoding *O*-acetyltransferase (Verma et al. 1991). *O*-acetyltransferase adds an *O*-acetyl group to the rhamnose III of the O-antigen, responsible for the 3b serotype. The O-antigen modification is one of the key virulence determinants of *S. flexneri* pathogenesis as it promotes the bacterial invasion and the evasion of innate immunity (West et al. 2005). Although the Sf6 bacteriophage has a highly mosaic genome of 39 kb (Casjens et al. 2004), SFL1520 was found to have only the intact *oac* gene and a few cryptic genes from Sf6. The upstream of Sf6 phage region in SFL1520 possesses several transposases and mobile genetic elements making this area a recombination hotspot. These findings suggest that the Sf6 phage had undergone several recombination events resulting in gene deletions ultimately leading to a defective or cryptic prophage as seen in many other bacterial pathogens (Campbell 1998; Canchaya et al. 2003). Besides the O-antigen modifying genes, the SFL1520 possessed SHI-2 PAI consisting of genes encoding the aerobactin iron transport system (Vokes et al. 1999). The SFL1520 chromosome also possessed seven *ipaH* genes as with other *S. flexneri* serotypes (Ashida et al. 2007). These genes are unique to *Shigella* and enteroinvasive *E.coli* and present in multicopy on both the invasion plasmid and the chromosome (Venkatesan et al. 1991). The *ipaH* genes possess a novel E3 ligase domain at the C-terminus and a series of leucine-rich repeats at the N-terminus capable of subverting the host’s ubiquitination pathway and have homologues in many species of bacterial pathogens (Quezada et al. 2009). The 20.4-kb putative PAI in SFL1520 consists of antibiotic resistance genes *tetDCAR, cat, dhfrI*, and *ant1* flanked by transposases. This multidrug resistance gene cassette is identical but inversely oriented to that of the multidrug resistance region of the 66-kb PAI, first identified in *S. flexneri* serotype 2a strain YSH6000 and was referred to as the SRL PAI (Luck et al. 2001). The identical multidrug resistance cassette was also identified in *S. flexneri* serotype Xv strain 2002017 (Ye et al. 2010). The antibiotic resistance gene cassette of this island is flanked by transposases on either side and had some uncharacterized genes of plasmid origin. Interestingly, these multidrug resistance genes with similar arrangement have also recently been reported in an *E. coli* plasmid, pRSB225 (Wibberg et al. 2013). Furthermore, the multidrug resistance cassette is capable of independent excision from the chromosome suggesting its ability to recombine with other genomes (Turner et al. 2001). The high prevalence of IS elements in *S. flexneri* genome (7% in case of the SFL1520 chromosome) makes this *S. flexneri* genome highly susceptible to recombination. The acquisition of such multidrug resistance genes by *S. flexneri* has become a challenge in developing countries, particularly in areas of limited resources for disease surveillance and management. The SFL1520 lacks SHI-1 PAI, which is not uncommon, being absent from many *S. flexneri* genomes (Al-Hasani et al. 2001; Nie et al. 2006; Baker et al. 2014). Experimental infection studies have shown that genes in SHI-1 PAI are responsible for initial colonization and fluid accumulation in the gut resulting in characteristic watery diarrhea in *Shigella* infection (Fasano et al. 1997; Henderson et al. 1999).

Analysis of the core and accessory genes of 11 complete *S. flexneri* genomes revealed a pangenome with 6,056 homologous groups, about a half of them were common to all of the *S. flexneri* strains. SFL1520 was found to have the largest number of unique genes (201) of the strains compared, despite the frequency of accessory genes being similar to all other strains. However, the pangenome size would continue to grow and the number of unique genes within a genome under comparison will decrease as additional *S. flexneri* genomes are sequenced (Parajuli et al. 2017). It is suspected that a significant factor of this accessory genome diversification is IS elements as a correlation was observed in *S. flexneri* strains between the number of IS elements and the frequency of unique genes. Although the overall base composition in SFL1520 is similar to other *S. flexneri* strains (50.9%), there was variable GC content throughout the SFL1520 bacterial chromosome. This further indicates the extent of horizontal gene acquisition as the recently acquired genes reflect the DNA composition of the donor genome (Lawrence and Ochman 1997). Interestingly, the overall GC content and genome size of all *S. flexneri* strains remain constant, suggesting the robustness of these bacterial genomes in adapting to evolutionary pressures. It will be interesting to find out why these acquired genes have been retained in the bacteria with the cost to maintain the genome size; in particular, to determine whether these genes provide a survival benefit to the bacteria and potentially impact pathogenesis and virulence.

The genome-wide alignment of *S. flexneri* genomes highlighted that many genomic blocks are either shuffled or inverted in SFL1520. Despite the accessory genome and synteny of genomic blocks of SFL1520 being identified as different compared with other strains, phylogenetic analysis based on the core genes found SFL1520 to be very similar to other *S. flexneri* strains. These results, therefore, suggest that SFL1520 might have undergone significant horizontal gene acquisition in a relatively short period.

The *S. flexneri* SFL1520 genome has several genes of bacteriophage and plasmid origin. These genes were most likely acquired horizontally by means of bacteriophage integration and transposon-mediated horizontal gene acquisition. These acquired genes had shaped the major virulence signatures of SFL1520, including serotype conversion and multidrug resistance. Currently, many of the unique genes identified in SFL1520 and other *S. flexneri* strains are hypothetical and, therefore, invite further analysis to uncover the function of these hypothetical genes. Further understanding of variation between strains might provide a platform to unfold more about differences in pathogenesis, host-interaction and, consequently, health outcomes.

## Supplementary Material

Supplementary data are available at *Genome Biology and Evolution* online.

## Supplementary Material

Supplementary Data

## Notes

**Data deposition:** This project has been deposited at GenBank under bioproject PRJNA492813 with complete genome sequence accession number CP032513.
