# Supplementary material for: Genome Analysis of Shigella flexneri Serotype 3b Strain SFL1520 Reveals Significant Horizontal Gene Acquisitions Including a Multidrug Resistance Cassette
Source: Genome Biol Evol. 2019 Feb 1;11(3):776–85. doi: 10.1093/gbe/evz026 (PMC6424224; doi:10.1093/gbe/evz026)
Supplement: Supplementary Data [file evz026_supp.zip › Supplimentary Figure S1.docx]

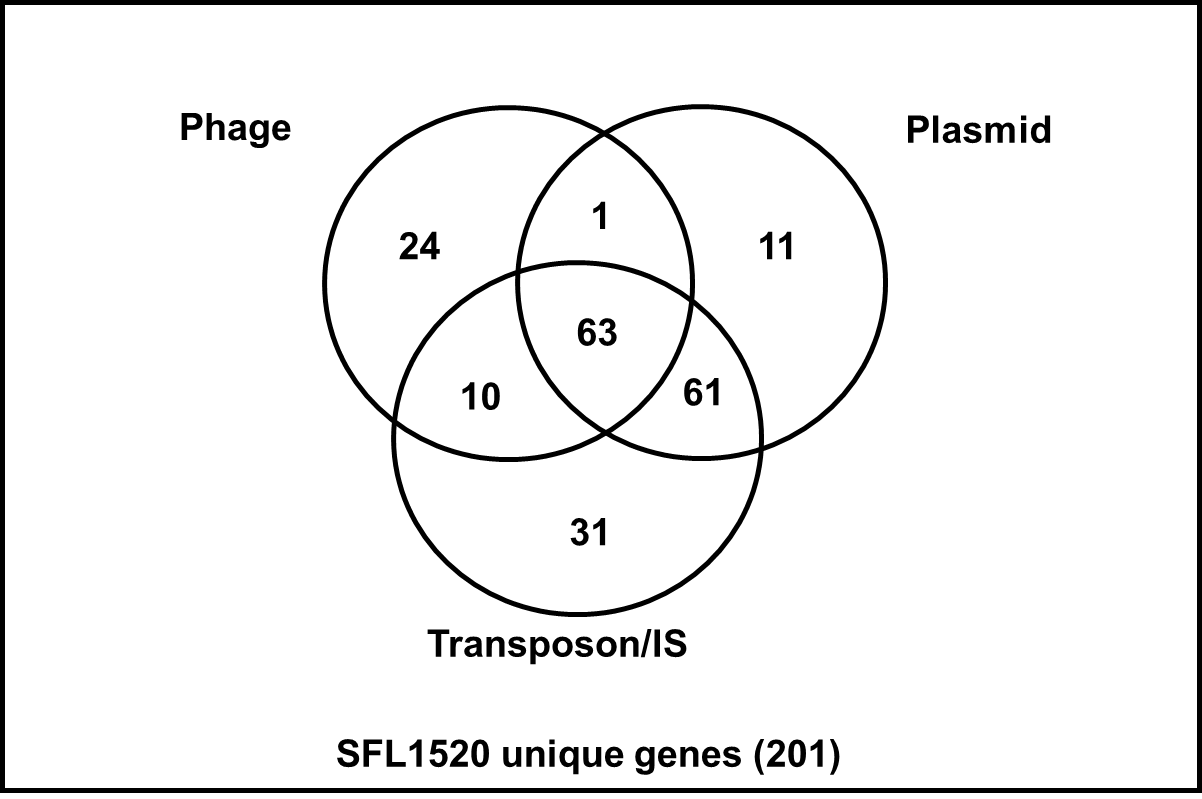


**Supplementary Figure S1: Potential source of unique genes in SFL1520**. The numbers in the Venn diagram indicate blast matches using ≥ 80 % sequence identity and ≥ 90% query coverage.
