# Supplementary material for: Genome Analysis of Shigella flexneri Serotype 3b Strain SFL1520 Reveals Significant Horizontal Gene Acquisitions Including a Multidrug Resistance Cassette
Source: Genome Biol Evol. 2019 Feb 1;11(3):776–85. doi: 10.1093/gbe/evz026 (PMC6424224; doi:10.1093/gbe/evz026)
Supplement: Supplementary Data [file evz026_supp.zip › Supplimentary Figure S2.docx]

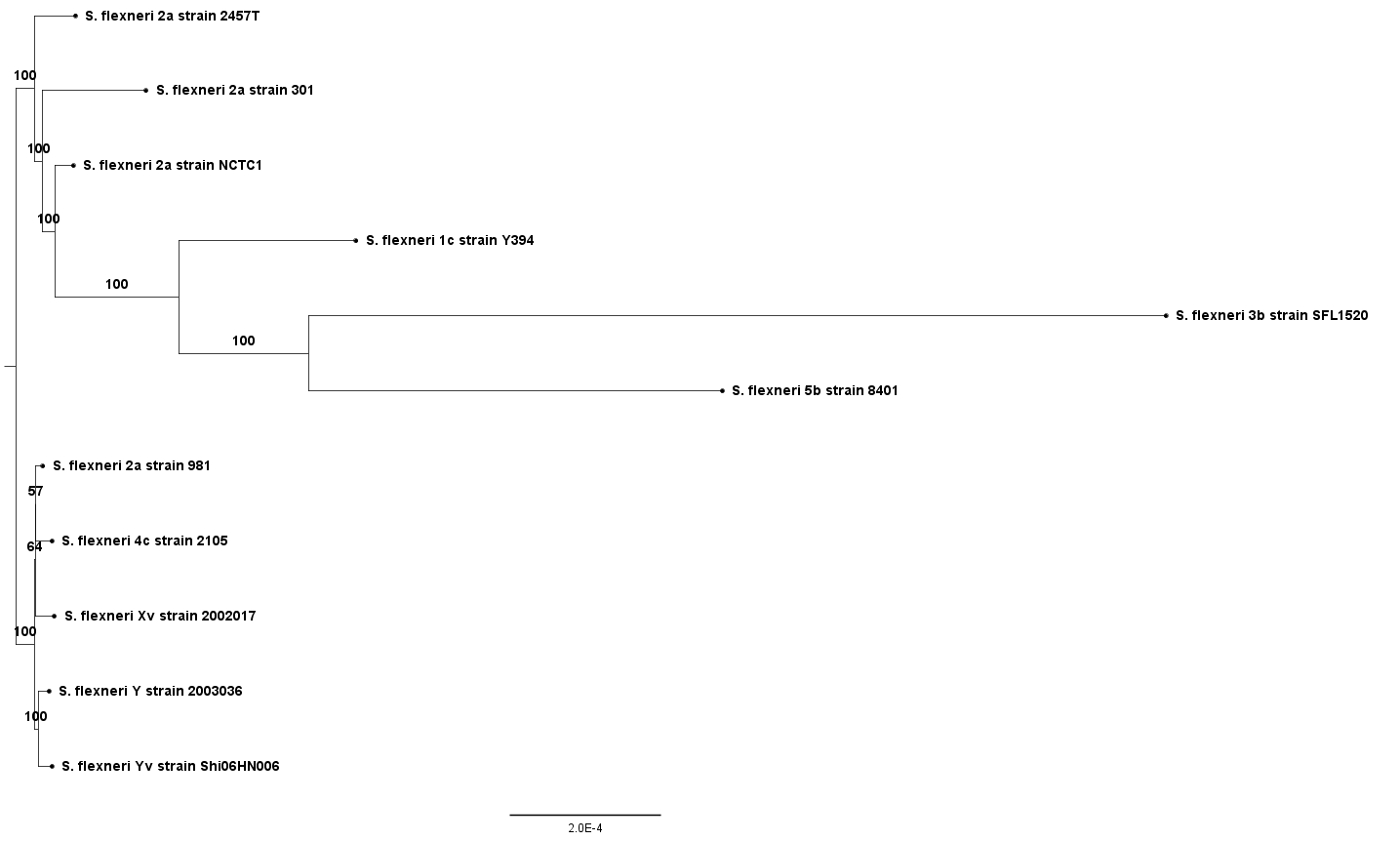


**Supplementary Figure S2: Phylogenetic tree using core genes of *Shigella flexneri* genomes***.* The maximum likelihood tree of eleven *S. flexneri* complete genomes based on alignment of 2,803 core genes. The numbers indicate the bootstrap support values for 1,000 pseudo-replicates. The scale bar represents substitution per site.
