# Supplementary material for: Genome Analysis of Shigella flexneri Serotype 3b Strain SFL1520 Reveals Significant Horizontal Gene Acquisitions Including a Multidrug Resistance Cassette
Source: Genome Biol Evol. 2019 Feb 1;11(3):776–85. doi: 10.1093/gbe/evz026 (PMC6424224; doi:10.1093/gbe/evz026)
Supplement: Supplementary Data [file evz026_supp.zip › Supplimentary Table S1.docx]

**Supplementary Table S1: List of complete genomes and their accession numbers used for comparative genomics.**

| S.N | Bacterial species | Accession Numbers |
| --- | --- | --- |
| 1 | *Escherichia coli* O157:H7 strain WS4202 | CP012802.1 |
| 2 | *Escherichia coli* strain K-12 substr. MG1655 | NC_000913.3 |
| 3 | *Klebsiella pneumonae* strain MGH 78578 | NC_009648.1 |
| 4 | Salmonella enteric*a* strain SL1344 | FQ312003.1 |
| 5 | *Shigella boydii* 4 strain Sb227 | CP000036.1 |
| 6 | *Shigella boydii* strain ATCC 9210 | CP011511.1 |
| 7 | *Shigella dysenteriae* strain 1617 | CP006736.1 |
| 8 | *Shigella dysenteriae* strain Sd197 | CP000034.1 |
| 9 | *Shigella flexneri* 1c strain Y394 | CP020753.1 |
| 10 | *Shigella flexneri* 2a strain 2457T | AE014073.1 |
| 11 | *Shigella flexneri* 2a strain 301 | AE005674.2 |
| 12 | *Shigella flexneri* 2a strain 981 | CP012137.1 |
| 13 | *Shigella flexneri* 2a strain NCTC1 | LM651928.1 |
| 14 | *Shigella flexneri* 4c strain 1205 | CP012140.1 |
| 15 | *Shigella flexneri* 5b strain 8401 | CP000266.1 |
| 16 | *Shigella flexneri* Xv 2002017 | CP001383.1 |
| 17 | *Shigella flexneri* Y strain 2003036 | CP004056.1 |
| 18 | *Shigella flexneri* Yv strain Shi06HN006 | CP004057.1 |
| 19 | *Shigella sonnei* strain FDAARGOS_90 | CP014099.1 |
| 20 | *Shigella sonnei* strain Ss046 | CP000038.1 |
